# What would be the benefits of a collaboration between psychoanalysis and cognitive neuroscience? The opinion of a neuroscientist

**DOI:** 10.3389/fnhum.2013.00475

**Published:** 2013-08-23

**Authors:** Perrine Ruby

**Affiliations:** ^1^Brain Dynamics and Cognition Team, Lyon Neuroscience Research Center (CRNL), INSERMCNRS, Lyon, France; ^2^University Lyon 1Lyon, France; ^3^Department of Psychology, University of SwanseaSwansea, UK

**Keywords:** unconscious memory of experienced events, personal meaning, subject's history, affective unconscious, self

## Context of development and guiding principles of psychoanalysis and cognitive neuroscience

Psychoanalysis was conceived and developed for clinical purposes at the beginning of the 20th century. Freud's main goal was to treat neurotic patients and psychotic patients. As a consequence of this great enterprise he developed a theory to explain the functioning of the human mind. A critical contribution of his work was the theory of the unconscious and the proposal that even if unconscious, a representation can influence a subject's behavior. Freud believed that unconscious thoughts and feelings may cause a patient to experience life difficulties and/or maladjustments. He proposed that the process of freeing unconscious thoughts could help a patient gain insight into and ultimately improve his/her situation. Therefore, Freud developed techniques to decode unconscious images, and to free them through patient insight (e.g., Freud, 1901).

Cognitive neuroscience is a rather young discipline. It was developed in the 1980's and has been strongly linked to the advancement of neuroimaging techniques (mainly positron emission tomography, PET, and functional magnetic resonance imaging, fMRI). The main goal of this discipline is to understand the functioning of the human brain/psyche. A consequence of this great enterprise has been, and hopefully will be, clinical applications. Cognitive neuroscience is fundamentally interested in processes/effects which can be found in several subjects rather than in the specific functioning of single subjects. Of importance, at the moment, this discipline does not provide a consensual and comprehensive theory of the human mind. It has, nevertheless, demonstrated that neuroscientific results can help to shape psychological theories or to disentangle between psychological theories (e.g., Henson, 2005; Poldrack, 2006; Legrand and Ruby, 2009).

## Pitfalls and gaps in the two disciplines showing the need for a bilateral collaboration

Psychoanalysis is considered by many scientists as an unscientific, an thus untrustworthy discipline. Psychoanalysis needs then to improve its credibility. A collaboration with a scientific discipline such as cognitive neuroscience could certainly help to achieve this aim. In addition, at the theoretical level, one cannot exclude that the investigation of the neurophysiological correlates of psychoanalytical concepts (e.g., Kaplan-Solms and Solms, 2000) could result in a better understanding of the links between some psychoanalytical concepts, as neurophysiological results have already resulted in a better understanding of concepts from experimental psychology (e.g., Henson, 2005; Poldrack, 2006; Legrand and Ruby, 2009).

A current weakness of cognitive neuroscience is to ignore some important brain/mind properties/characteristics, such as (1) what is important to the individual subjects, or in other words the notion of “meaning” in the Freudian sense, (2) the subject's history and preferences, (3) the notion of affective unconscious which is closely related to the Freudian unconscious, (4) the unconscious memory of experienced events (UMEE), which refers to the memory of an episode or a scene that cannot be consciously recollected. All of these issues are at the core of our intimate mind; they are the foundation of our identity. One cannot expect to propose a comprehensive theory of the human mind if neglecting these issues. A collaboration with psychoanalysis, which could be considered as a “science” of singularity, appears then providential to help finding solutions to address private issues in cognitive neuroscience (Ruby, 2011).

## What would be the benefits of a collaboration? the case of UMEE

### UMEE are neglected in cognitive neuroscience

In cognitive neuroscience, the dominant theory of memory states that long-term memory can be either explicit or implicit. These two types of memory are often referred to as declarative (composed of episodic and semantic memory) and procedural memory or the “knowing what” and the “knowing how.” This well-used way of naming explicit and implicit memories show that the notion of “knowing what implicitly/unconsciously” is not emphasized at the theoretical level. It is also true at the experimental level. The issue of UMEE in particular has been barely investigated in cognitive neuroscience. Rather, this concept is often ignored and possibly denied which has important consequences on the interpretation of results and thus theories of the brain/mind.

The consideration of UMEE has been lacking for example in research investigating memory and the self, as illustrated by the article of Klein and Gangi in the Annals of the New York academy of sciences (Klein and Gangi, 2010). In this study, the authors aimed to better understand the link between the different types of self-related memory systems, by investigating the representation of self-personality traits in patients with amnesia. They report results showing that some patients with episodic amnesia (with traumatic or developmental etiologies) can, despite their amnesia, update the representation of their own personality traits. For Klein and Gangi, these cases showed that episodic and semantic memory systems were separate and independent. Interestingly, they also considered at some point the possibility that UMEE may participate in the updating of personality traits since they wrote “K.C. not only had access to semantic knowledge of his own personality traits, but he was also able to acquire new knowledge about his personality. Yet this updating occurred without his being able to episodically recollect any information about the behavioral events on which this updating presumably was based.” Unfortunately, the authors did not develop this point and did not discuss the hypothesis of the updating of personality traits based on the UMEE. However, according to their results, one cannot exclude this possibility. How could one otherwise explain their results? The most likely hypothesis is that a semantic representation of one's own personality is elaborated using memories (conscious or unconscious) of past episodes of one's own life, and especially episodes involving human interactions. Klein and Gangi did not provide an alternative explanation but argued against this hypothesis by stating that they “devoted a substantial amount of [their] research (consisting in multiple methods—for example, priming techniques, transfer appropriate processing, the method of reversed association^39, 55^) to show that exemplar-based self-knowledge is not activated (consciously or unconsciously) when participants perform semantic judgments about the self.^29, 35^.” However, these results do not exclude the possibility that UMEE participate in the formation of semantic knowledge even if it is not activated during semantic tasks.

UMEE is also barely considered in the field of dream research. Memory (be it conscious or not) is the main source of information available during sleep. Previous studies have looked for episodic recall in the content of dreams (e.g., Fosse et al., 2003), but to my knowledge, no studies have investigated whether UMEE show up in dream content or not. Investigating autobiographical memory in the elderly, Grenier et al. (2005) showed that dreams could bring back very old memories and especially memories from adolescence. Interestingly, these results were not explained by a recent reminiscence of these remote memories because “the participants indicated that to the best of their knowledge, they had not thought of or talked about the different elements experienced in their dreams since the time of the original experience.” This type of memory may be closely related to unconscious memory (repressed or not) because according to the participants, the episode recalled in the dream had not reached consciousness since this episode actually happened. It seems thus fairly possible that dreams could bring unconscious memories or representations to consciousness, but this has not yet been tested.

### UMEE are at the core of psychoanalytic practices

Even if they are difficult to uncover and confirm, UMEE does exist. Here is a tragic example from the French news from the winter of 2012 (e.g., Sud Ouest 29/02/2012; Le Monde 03/03/2012). A women living in Lyon, France (Zahia H.) recalled the memory of being raped and assaulted 37 years prior, in 1973, upon awakening from general anesthesia in 2010. The surgery (one of many previous surgeries) was indicated to treat disabilities caused by this aggression. Thirty seven years ago, Zahia awoke from a coma induced by the episode and had no memory of the rape and the head injury which she had sustained. Zahia's mother decided not to tell her what had occurred. Thirty seven years after, when Zahia recalled the memory of this tragic episode her mother was dead.

This case demonstrates the existence of unconscious memories of important experiences (the facts can be verified). One can easily intuit reading this story, that memories, even if implicit/unconscious, may influence behavior (for example, unexplained fear in particular situations) and play an important role in a subject's life, as hypothesized by Freud. In support of this single case, Mitchell (2006) managed to produce experimental results showing the existence of long-lasting unconscious memory of images. He demonstrated that pictures presented 1–3 s could induce a priming effect 17 years after presentation, even in subjects who reported no conscious recollection of their participation in the original laboratory session.

Currently, cognitive neuroscience cannot easily explain the recovery of a memory 30 or 40 years later. By contrast, this phenomenon can be explained from a psychoanalytic perspective (When I previously described Zahia's case in a conference, René Roussillon, psychoanalyst and professor of psychology at the University of Lyon, predicted that the mother of Zahia was dead when she recovered the memory of the rape even though I did not mention this fact). This comes as no surprise since the core of psychoanalytic practice is centered on unconscious memories or representations that induce life difficulties and/or maladjustment and on the means to free them.

### What would be the benefit of a collaboration between psychoanalysts and neuroscientists on UMEE

On the one hand, UMEE is a component that can be easily incorporated into a theoretical model of memory in cognitive neuroscience; on the other hand, this type of memory plays a central role in psychoanalysis since conflicts and trauma may lead (via repression or not) to the creation of UMEE. Therefore, the UMEE (resulting from repression or not) could be a strong convergence point between psychoanalysis and cognitive neuroscience, which may help to built bridges between the two disciplines. Below, possible benefits of a collaboration between psychoanalysis and cognitive neuroscience on UMEE.

#### New hypotheses on the functioning of memory systems in cognitive neuroscience

Adding a psychoanalytical perspective to reflections on cognitive neuroscience experiments may result in new hypotheses. This can be illustrated by the two examples described above. First, the hypothesis of the possible participation of UMEE in the formation of semantic knowledge about the self derives from a psychoanalytical perspective on the functioning of the mind/brain. Testing this hypothesis would help to better understand the formation of the semantic representation of one's personality traits, which according to Klein and Gangi (2010), is still a mystery: “Of the systems of self we have examined, the semantic self-knowledge system seems the most resilient in the face of the cognitive chaos resulting from developmental and/or environmental damage to the brain. This is both an empirical fact and a mystery for which we have, at present, no explanation.” Second, according to Freud (e.g., Freud, 1901), unconscious representation may surface during dreams. Collaboration with psychoanalysts could assist in the design of experiments to test this hypothesis. For example, one could try to use the free association method to test whether free association initiated from the words of a dream would lead to UMEE. The results would help to elucidate whether dreams are indeed the royal road to the unconscious and could also have important implications for theories of sleep, dreams and memory.

#### New paradigms to test unexplored issues in cognitive neuroscience

The absence of consideration of the UMEE in cognitive neuroscience is certainly due to the difficulty in investigating such private issue using an experimental approach. Psychoanalysts who work to make unconscious representations conscious may be helpful in developing methods to address this issue in cognitive neuroscience. Previous interdisciplinary work proves that novel paradigms can enable a scientific investigation of concepts that are seemingly impossible to explore. For example, Howard Shevrin, psychoanalyst and Professor of Psychology at The University of Michigan, developed shrewd paradigms to investigate unconscious processes at the experimental level. Using event related potentials and a free association method, his team identified neurophysiologic markers of subliminal perceptions and showed that subliminal images were processed in a complex and associative way (Shevrin and Fritzler, 1968). Using similar means (mild electric shock presented 800 ms after images or words presented subliminally) he also demonstrated that aversive conditioning can occur unconsciously (Shevrin, 2001). These results had a great impact on the neuroscientific community and precipitated an interest in unconscious processes.

#### Optimize therapeutic means in the psychoanalytic practice

The investigation of UMEE in cognitive neuroscience should result in a better understanding of the context of formation (repression or not) and the neurophysiological basis of UMEE (possibly dependent on the context of formation). Theoretically, this should help to optimize the therapeutic means to act on or free such memories.

## Conclusion

Even if difficult, unconscious processes, private issues (preferences, history, what matters for a subject.) and, more generally, psychoanalytic concepts have to be addressed at the experimental level to achieve a comprehensive theory of the human mind. Another great benefit of such an endeavor would be to provide objective arguments and allow a constructive and scientific debate about whether Freudian or psychoanalytical concepts are plausible and useful. In other words, a collaboration between psychoanalysis and cognitive neuroscience may be the best way to escape from the sterile and counterproductive hostility between the disciplines and to move forward together in order to benefit science and medical practices.
